# Bacterial etiology and antimicrobial resistance patterns of pediatric UTIs in the West bank, Palestine: a cross-sectional study

**DOI:** 10.1186/s12887-025-06054-0

**Published:** 2025-10-02

**Authors:** Tala Amleh, Aseel Salah, Reem Hamayel, Bayan A. Ibrahim, Hamza Sayeh, Suha Hamshari, Mohammad Qadi

**Affiliations:** 1 Department of Medicine, Faculty of Medicine and Allied Medical Sciences, An-Najah National University, P.O. Box. 7, Nablus, State of Palestine; 2 Department of Pediatrics, Rafedia Governmental Hospital, Ministry of Health, Nablus, State of Palestine; 3 Department of Biomedical Sciences and Basic Clinical Skills, Faculty of Medicine and Allied Medical Sciences, An-Najah National University, P.O. Box. 7, Nablus, State of Palestine

**Keywords:** Urinary tract infection (UTI), Pediatric infectious diseases, Antimicrobial resistance (AMR), Multidrug-resistant (MDR) pathogens, Infection prevention and control, Antibiotic stewardship, Public health resilience, Healthcare sustainability in palestine.

## Abstract

**Introduction:**

Urinary tract infections (UTIs) are among the most common bacterial infections in children. This study aimed to investigate the distribution of bacterial uropathogens and their antimicrobial resistance patterns among hospitalized pediatric patients with UTIs in governmental hospitals across the West Bank, Palestine. The findings are intended to support evidence-based empirical treatment strategies and inform local antimicrobial stewardship efforts.

**Methods:**

This is a cross-sectional study, analyzed urine culture results from pediatric patients with UTI symptoms admitted to governmental hospitals across three regions of the West Bank in 2021–2022, using data from the Ministry of Health’s electronic database.

**Results:**

3,949 positive urine culture results were analyzed; 58.5% of the total were aged ≥ 2 years, and 2883 were females (73%), with Gram-negative bacteria accounting for 87.1%. The most common pathogen was *Escherichia coli* (61.7%). The prevalence of multidrug-resistant (MDR) bacteria was 34.3%. Among Gram-negative isolates, 34.5% were MDR, 33.1% were extended-spectrum beta-lactamase (ESBL) producers, and 3.3% were carbapenem-resistant *Enterobacterales* (CRE). The highest MDR percentage was in *Proteus* spp. (50.6%) followed by *Klebsiella* spp. (45.4%), and *E. coli* (32.6%). According to matching with the empirical antibiotics, Ampicillin shows 16.8%, Ceftriaxone shows 49.5%, and Cefotaxime shows 51.9%.

**Conclusion:**

Our findings highlight the high prevalence of MDR bacteria among pediatric UTI patients in the West Bank. *E. coli* and *Klebsiella* spp. were the predominant pathogens, showing considerable resistance to commonly used antibiotics. These results underscore the need to reassess empirical UTI treatment regimens, conduct routine antibiogram monitoring, and implement coordinated infection control and antibiotic stewardship programs across the West Bank, Palestine.

**Supplementary Information:**

The online version contains supplementary material available at 10.1186/s12887-025-06054-0.

## Introduction

Urinary tract infections (UTIs) represent a significant health concern in the pediatric population, contributing to notable morbidity and, if untreated, even mortality. These infections commonly present with symptoms such as fever, malodorous urine, hematuria, abdominal pain, or urinary incontinence, while older children may exhibit dysuria, frequency, or urgency of urination. Infants, particularly male infants under six months and uncircumcised males under 12 months, along with female infants under one year, are at higher risk of UTIs [1–3]. Gram-negative bacteria, especially*Escherichia coli*, are the leading pathogens in pediatric UTIs, accounting for 80–90% of cases. Other causative organisms include *Klebsiella* spp., *Proteus* spp., and *Pseudomonas*spp., with pathogen distribution varying by age, sex, and underlying health conditions [4, 5].

However, the growing prevalence of antimicrobial resistance (AMR) among UTI pathogens poses a substantial challenge to effective treatment [6–8]. Pathogens showed varying patterns of resistance [5, 7–9]. Of particular concern are organisms displaying multidrug resistance (MDR), extensive drug resistance (XDR), and extended-spectrum β-lactamase (ESBL) production.*Enterobacterales* are the main producers of ESBL enzymes, which break down and help resist penicillins, cephalosporins (like ceftriaxone, ceftazidime, and cefotaxime), and monobactams (like aztreonam), but they do not affect cephamycins (like cefoxitin) or carbapenems. Organizations such as the European Committee on Antimicrobial Susceptibility Testing (EUCAST) and the Clinical and Laboratory Standards Institute (CLSI) standardize susceptibility testing and ESBL detection. They offer confirmatory testing techniques and interpretive criteria (M100, VET01/VS01 https://clsi.org/shop/standards/vet01/). According to consensus by the European Centre for Disease Prevention and Control (ECDC) and the US Centers for Disease Control and Prevention (CDC), MDR is broadly defined as non-susceptibility to at least one agent in three or more antimicrobial categories relevant to the organism. XDR represents non-susceptibility to at least one antimicrobial in all but two or fewer antimicrobial drug categories (i.e., susceptibility to only one or two categories) [10].

In Palestine, several studies have highlighted the increasing prevalence of antimicrobial-resistant pathogens. An earlier study conducted in Gaza in 2001, which examined urine cultures from adult female patients with UTIs, reported that only 3.3% of 300 *E. coli*isolates were ESBL producers [11]. In contrast, a later study from Gaza in 2013, which analyzed clinical specimens from various infection sites over a 3-month period, found significantly higher ESBL production rates: 59.3% among*Klebsiella pneumoniae* and 39.1% among *E. coli*isolates [12], indicating a notable rise in resistance over time. Additionally, a large retrospective study conducted in the West Bank between 2019 and 2021 analyzed resistance patterns among neonates with bacteremia across three governmental hospitals located in the northern, central, and southern regions. The findings revealed alarming resistance rates: 29% of all isolated pathogens were classified as MDR, with MDR detected in 87% of*Klebsiella* spp. and 52.2% of *E. coli*isolates. These data underscore the growing threat of MDR and ESBL-producing organisms across both the Gaza Strip and the West Bank, emphasizing the urgent need for continuous surveillance and updated local resistance data to guide empirical treatment strategies [13].

International treatment guidelines for uncomplicated UTIs recommend antibiotics like nitrofurantoin, trimethoprim-sulfamethoxazole, and fosfomycin, but their efficacy is increasingly compromised by AMR. For example, *E. coli*has shown high resistance rates to commonly used antibiotics such as ampicillin (66.6%) [14]. The emergence of MDR pathogens not only complicates clinical management but also increases healthcare costs due to prolonged hospital stays and the need for more complex treatments [6, 15].

To combat these challenges, continuous surveillance of antimicrobial susceptibility patterns is essential. This surveillance informs appropriate antibiotic prescribing and guides updates to treatment protocols. Additionally, implementing robust infection prevention measures, such as proper hygiene practices and timely diagnosis and treatment, can help reduce the burden of UTIs in pediatric populations [8, 9].

This study was conducted to analyze the prevalence of bacterial uropathogens and their antimicrobial resistance profiles among hospitalized pediatric patients with UTIs across governmental hospitals in the West Bank, Palestine, between 2021 and 2022. By identifying the predominant pathogens and their resistance patterns, the study aims to support evidence-based empirical antibiotic selection based on local data and contribute to global efforts in promoting rational antibiotic use.

## Methods

### Study design, setting, population

#### Data collection procedure, inclusion, and exclusion criteria

**Fig. 1:**
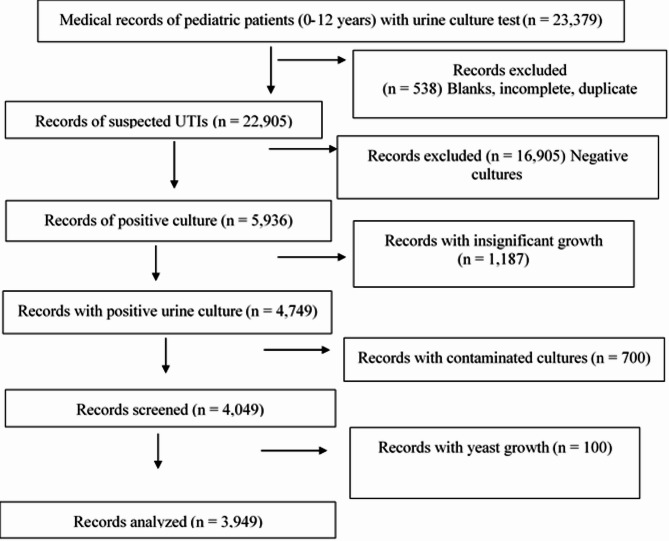
Flow chart of the study procedure for participant selection

Overall, 23,379 urine culture tests were collected. We excluded tests linked to blank, incomplete, or duplicated medical records. Negative culture results and insignificant growth were also excluded (cultures with colony count of < 10^5^ colony-forming unit/ml of urine for patients > 2 years old, and less than < 10^3^colony-forming unit/ml for patients < 2 years old). The variation in significant growth limits for both age groups is due to the different sampling techniques, where catheter sampling was used for patients younger than 2 years and midstream catch urine for those older than 2 years. Contaminated cultures (growth of ≥ 3 organisms in a urine sample) [16] and culture records with yeast growth were also excluded. The total excluded medical records were 19,430; the final number of urine culture records included in this study was 3949, as shown in Fig.1.

#### Bacterial identification and antimicrobial susceptibility testing

All microbiological data were collected and analyzed in the microbiology laboratories of the participating hospitals. Bacterial identification and antimicrobial susceptibility testing were performed using the VITEK^®^ 2 Compact system (bioMérieux, France), employing GN and GP identification and susceptibility cards appropriate for the tested organisms. The system automatically interprets minimum inhibitory concentrations (MICs) and generates susceptibility results based on the Clinical and Laboratory Standards Institute (CLSI) guidelines, ensuring standardized and reliable reporting.

For isolates suspected of ESBL production by the VITEK system, an ESBL confirmatory test was performed based on the detection of synergy between third-generation cephalosporins: cefotaxime (30 µg) and ceftazidime (30 µg) and amoxicillin/clavulanic acid (20/10 µg). Confirmation was carried out according to CLSI recommendations.

#### Microbiological definitions

A positive urine culture was considered when it showed growth of a concentration of ≥ 10⁵ colony-forming units per milliliter (CFU/mL) of a single organism following standard pediatric guidelines based on CLSI criteria. Multidrug-resistant organisms were defined as isolates resistant to at least one agent in three or more antimicrobial categories, based on the international consensus definition (Magiorakos et al., 2012). Extended-spectrum beta-lactamase-producing organisms were identified by resistance to third-generation cephalosporins, with confirmation based on CLSI criteria. Carbapenem-resistant Enterobacteriaceae were defined as species with resistance to at least one carbapenem antibiotic (e.g., meropenem or imipenem), in line with CLSI breakpoints.

#### Ethical coonnsiderati

Ethical approval (Ref: Med. Sept. 2023/22) was obtained from the Institutional Review Board (IRB) at An-Najah National University in Palestine. Additional approval to access medical files from the Palestinian Ministry of Health was obtained. The requirement for informed consent to participate, including for participants under the age of 16, was waived by the IRB, as the study involved retrospective data collection and posed minimal risk to participants. All obtained original data were treated confidentially and were available only to the research team. Codes were used instead of names to ensure confidentiality; therefore, data were analyzed anonymously.

#### Statistical analyses

The collected data were summarized and analyzed using SPSS Version 25.0 and Microsoft Excel 2017. We assessed the most common uropathogens causing UTIs and antibiotic susceptibility patterns with descriptive statistics. Univariate analysis (Chi-square) was used to examine the association between the dependent variable (UTIs) and independent variables (causative organisms and their AMS). A *p* < 0.05 was considered significant.

## Results

A total of 3,949 pediatric patients with positive bacterial urine cultures confirming UTIs were included in the study, 58.5% of the total were aged ≥ 2 years (*n* = 2311), while 41.5% were < 2 years (*n* = 1638). 2883 were females (73%), and 1066 were males (27%). Corresponding to 2022, 1900 pediatric UTI positive cultures were recorded (48.1%) compared to 51.9% (*n* = 2049) in 2021. Regarding the hospital distribution, 1893 admitted cases were in the north (47.9%), 1092 in the middle (27.7%), and 964 in the south (24.4%), as shown in Table 1.

**Table 1:** Background information of the pediatric patients admitted with UTI (*n* = 3,949)

Variable	Category	*n* (%)
Age (years)		
	< 2	1638 (41.5)
	≥ 2	2311 (58.5)
Gender		
	Male	1066 (27.0)
	Female	2883 (73.0)
Study Year		
	2021	2049 (51.9)
	2022	1900 (48.1)
Hospital		
	Middle region hospital	1092 (27.7)
	Northern region hospital	1893 (47.9)
	Southern region hospital	964 (24.4)

The predominant causative UTI pathogens were Gram-negative bacteria with 3440 cases (87.1%). *E. coli* was vastly identified with statistically significant (61.7%, *n* = 2438, p-value < 0.001), which was higher in pediatrics aged ≥ 2 years (69.6%, *n* = 1609, p-value < 0.001), and in females (69.8%, *n* = 2014, p-value < 0.001). *Klebsiella* spp. Followed *E. coli* with 601 cases (15.2%), notably higher in pediatric aged < 2 years (26% vs. 7.6%) and in males (26.5% vs. 11.1%) (p-value < 0.001). Other Gram-negative pathogens included *Pseudomonas* spp. (2.8%), *Proteus* spp. (2.1%), and *Enterobacter* spp. (1.7%).

Gram-positive bacteria were detected in 12.9% of culture-positive UTI cases included in this study; among them, *Enterococcus* spp. is the most common (5.9%), followed by Coagulase Negative was the most common (5.9%), followed by Coagulase-negative *Staphylococcus* spp. (CoNS) (5.0%) and *Staphylococcus aureus* (1.1%). Gram-positive infections were significantly more frequent in males, particularly those caused by *Enterococcus* spp. (10.2% in males vs. 4.4% in females, p-value < 0.001) and CoNS (7.6% in males vs. 4.1% in females, *p* = 0.006), as shown in Table 2.

**Table 2:** Bacteriological profile of pediatric patients with culture-positive UTI and its distribution across age groups and genders (*n* = 3,949)

Microorganism	Frequency	Age(years)			Gender		
	*n*(%) 3949	< 21638	≥ 22311	P_Value	Male1066	Female2883	P_Value
Gram-negative	**3440(87.1)**	**1420(86.7)**	**2020(87.4)**		**853(80)**	**2587(89.7)**	
* E. coli*	2438(61.7)	829(50.6)	1609(69.6)	< 0.001	424(39.8)	2014(69.8)	< 0.001
* Klebsiella* spp.	601(15.2)	426(26)	175(7.6)	< 0.001	282(26.5)	319(11.1)	< 0.001
* Pseudomonas* spp.	112(2.8)	37(2.2)	75(3.2)	< 0.001	38(3.6)	74(2.6)	0.094
* Proteus spp.*	83(2.1)	21(1.3)	62(2.7)	< 0.001	25(2.2)	58(2)	0.517
* Enterobacter* spp.	66(1.7)	50(3.1)	16(0.7)	< 0.001	37(3.5)	29(1)	< 0.001
Others	140(3.6)	57(3.5)	83(3.6)		47(4.4)	93(3.2)	
Gram-positive	**509(12.9)**	**218(13.3)**	**291(12.6)**		**213(20)**	**296(10.3)**	
* Enterococcus* spp.	234(5.9)	134(8.2)	100(4.3)	< 0.001	108(10.2)	126(4.4)	< 0.001
CoNS	198(5.0)	54(3.3)	144(6.2)	< 0.001	81(7.6)	117(4.1)	0.006
* S. aureus*	44(1.1)	16(0.97)	28(1.2)	0.113	16(1.5)	28(0.94)	0.159
Others	33(0.9)	14(0.85)	19(0.9)		8(0.7)	25(0.86)	

Among Gram-negative bacteria, MDR was identified in 34.5% (*n* = 1187), with statistically significantly higher rates in pediatrics aged < 2 years (43.8%) vs. those who were ≥ 2 years (28%), and in males (48.7%) compared to females (29.8%) (p-value < 0.001). ESBL was observed in 33.1% of Gram-negative cases (*n* = 1,140), with a significantly higher percentage in those who were aged < 2 years (38.5%) and males (45.4%) (p-value < 0.001). CRE was detected in 3.3% (*n* = 115) of Gram-negative cases, with a significantly higher percentage in the age group < 2 years (6.5) and in males (8%) (p-value < 0.001). Extensively Drug-Resistant (XDR) was identified in 1.5% of Gram-negative isolates, predominantly in male children aged < 2 years, as shown in Table 3.

Concerning Gram-positive isolates (*n* = 509, 12.9%), MDR was detected in 33% (*n* = 168) of Gram-positive cases, with a significantly higher percentage in children aged ≥ 2 years (41.3%) and in males (36.6%) (p-value < 0.001). Methicillin-resistant Coagulase-negative staphylococci (MR-CoNS) accompanied 11.1% of Gram-positive isolates. XDR was detected in 15.5% of Gram-positive cases, as shown in Table 3.

**Table 3:** Bacterial resistance pattern

Microorganism	Frequency	Age(years)			Gender		
	*n* (%)**3**,**949**	< 21638	≥ 22311	P_Value	Male1066	Female2883	P_Value
Gram-negative	**3440(87.1)**	**1420**	**2020**		**853**	**2587**	
MDR	1187(34.5)	622(43.8)	565(28)	< 0.001	415(48.7)	772(29.8)	< 0.001
ESBL	1140(33.1)	547(38.5)	586(29)	< 0.001	387(45.4)	753(29)	< 0.001
CRE	115(3.3)	93(6.5)	22(1)	< 0.001	69(8)	46(1.8)	< 0.001
XDR	52 (1.5)	38(2.7)	14(0.7)	< 0.001	26(3)	26(1)	< 0.001
Gram-positive	**509(12.9)**	**291**	**218**		**213**	**296**	
MDR	168(33)	78(26.8)	90(41.3)	< 0.001	78(36.6)	90(30.4)	< 0.001
VRE	3(0.6)	1(0.3)	2(0.9)	< 0.001	0	3(1)	< 0.001
MRSA	20(3.9)	8(2.7)	12(5.5)	0.007	5(2.3)	15(5)	0.112
MR-CoNS	57(11.1)	24(8.2)	33(15.1)	0.053	29(13.6)	28(9.5)	< 0.001
XDR	79(15.5)	45(15.5)	34(15.6)	< 0.001	29(13.6)	50(16.9)	< 0.001

Among the 3949 cases of the study, the MDR percentage was 34.3% (*n* = 1355). In the case of Gram-negative bacteria, MDR was identified in 34.5% (*n* = 1187), the highest MDR percentage was in *Proteus* spp. (50.6%) followed by *Klebsiella* spp. (45.4%), *E. coli* (32.6%), and *Enterobacter* spp. (25.8%). XDR were identified in 3.34% of all isolates and 1.5% of Gram-negative isolates, mainly in *Klebsiella* spp. ESBL-producing Gram-negative isolates were seen in 33.1% of the cases, with the highest percentage in *Klebsiella* spp. (39.8%) and *E. coli* (36.7%). ESBL and CRE were found in 33.1% and 3.3%, respectively, of the Gram-negative cases, mostly in *Klebsiella* spp. (10.7%), followed by *Pseudomonas* spp. (6.2%), and *Enterobacter* spp. (6%).

Among gram-positive bacteria, MDR accounts 33.1%; the highest rates were observed in *S. aureus* (29.5%), *Enterococcus* spp. (27.4%) then CoNS (23.7%). XDR was significantly higher among Gram-positive isolates (15.7%) than Gram-negative (1.5%), notably in *Enterococcus* spp. (24%). vancomycin resistant *Enterococcus* (VRE) was detected in 1.3% of isolates, while methicillin-resistant *S. aureus (*MRSA) accounted for 45.5% of *S. aureus* isolates. MR-CoNS was identified in 28.7% of the total CoNS isolates, as shown in Table 4.

**Table 4:** Distribution of bacterial pattern of resistance among the isolated bacterial species

		MDR	XDR	ESBL	CRE	VRE	MRSA	MR-CoNS
Total n (%)		1355(34.3)	132(3.34)	1140(28.9)	115(2.9)	3(0.08)	20(0.5)	57(1.4)
Microorganism	Total n							
Gram-negative	**3440**	**1187 (34.5)**	**52(1.5)**	**1140(33.1)**	**115(3.3)**	**-**		**-**
* E. coli*	2438	796 (32.6)	6(0.2)	894(36.7)	11(0.5)	-		-
* Klebsiella* spp.	601	273 (45.4)	20(3.3)	239(39.8)	64(10.7)	-		-
* Pseudomonas* spp.	112	6 (5.3)	1(0.9)	0(0)	7(6.2)	-		-
* Proteus* spp.	83	42 (50.6)	0(0)	0(0)	2(2.4)	-		-
* Enterobacter* spp.	66	17 (25.8)	3(4.5)	0(0)	4(6)	-		-
Others	140	80 (57.1)	22(15.7)	0(0)	27(19.4)	-		-
Gram-positive	**509**	**168 (33)**	**80(15.7)**	**-**	**-**	**3(0.6)**	**20(3.9)**	**57(11.2)**
* Enterococcus* spp.	234	64 (27.4)	56(24)	-	-	3(1.3)	-	-
CoNS	198	47 (23.7)	2(1)	-	-	-	-	57(28.7)
* Staphylococcus aureus*	44	13 (29.5)	0(0)	-	-	-	20(45.5)	-
* Streptococcus agalactiae*	28	1(3.6)	18(64.3)	-	-	-	-	-
Others	5	1(20)	0(0)	-	-	-	-	-

The prevalence and statistical significance of antimicrobial resistance patterns among isolates from the three regional hospitals are summarized in Table 5. Of the 1,355 MDR isolates identified, the highest proportion was observed in the middle region (49.9%), followed by the south (28.4%) and the north (21.7%), with a statistically significant difference (*p* < 0.001). Among the 132 XDR cases, 44.7% were detected in the southern region, 33.3% in the middle, and 22% in the north (*p* < 0.001). Similarly, of the 1,140 ESBL-producing organisms, 53.4% were from the middle region, 28.2% from the south, and 18.4% from the north (*p* < 0.001). The distribution of 115 CRE isolates also showed a significant regional difference (*p* = 0.002), with the highest prevalence in the middle region (63.5%), followed by the south (20%) and the north (16.5%). Although the distribution of MRSA was not statistically significant (*p* = 0.231), other organisms such as MR-CoNS and VRE showed significant regional variation (*p* < 0.001).

**Table 5:** Distribution of Antimicrobial-Resistant organisms across hospitals in the north, middle, and South regions of West bank, Palestine

Microorganism	Total *n*		Hospital		*P*-value
		NORTH *n* (%)	MIDDLE *n* (%)	SOUTH *n* (%)	
MDR	1355	294(21.7)	676(49.9)	385(28.4)	< 0.001
XDR	132	29(22)	44(33.3)	59(44.7)	< 0.001
ESBL	1140	209(18.4)	609(53.4)	322(28.2)	< 0.001
CRE	115	19(16.5)	73(63.5)	23(20)	0.002
VRE	3	-	2(66.6)	1(33.4)	< 0.001
MRSA	20	9(45)	8(40)	3(15)	0.231
MR-CoNS	57	15(26.3)	4(7)	38(66.7)	< 0.001

According to matching with the used empirical antibiotics, Cefotaxime exhibited the highest rates, which reached 51.9%, mainly in the age group ≥ 2 years (57.3%) vs. age group < 2 (44.2%) and in females (56.8%) vs. males (38.6%) (p-value < 0.001).

Ceftriaxone matching was observed in 49.7% of all isolates, highest in the age group ≥ 2 (54.7%) years in comparison to 42.5% in < 2 years, with a statistically significant difference (p-value < 0.001). Additionally, there is a slightly higher difference between females (54.7%) to males (36.2%) (p-value < 0.001).

Ampicillin matching followed the same trend globally, with decreased matching rates in all isolates (16.8%), mainly in children < 2 years (14.3%) than in those ≥ 2 years (18.6%), and in males (13.3%) than in females (18.1%) (p-value = 0.014), as shown in Table 6.

**Table 6:** Matching with the empirical treatment

		Age			Gender		
Total n (%)		< 2	≥ 2	*P*_Value	Male	Female	*P*_Value
Total	**3949**	**1638**	**2311**		**1066**	**2883**	
Ampicillin	**664(16.8)**	234(14.3)	430(18.6)	< 0.001	142(13.3)	522(18.1)	0.014
Ceftriaxone	**1962(49.7)**	697(42.5)	1265(54.7)	< 0.001	386(36.2)	1576(54.7)	< 0.001
Cefotaxime	**2049(51.9)**	724(44.2)	1325(57.3)	< 0.001	412(38.6)	1637(56.8)	< 0.001

A detailed antibiotic susceptibility profile for the commonly tested antibiotics is listed in Supplementary Table S1.

## Discussion

This study conducted a comprehensive analysis of 3,949 positive urine culture records in the West Bank, Palestine. Gram-negative bacteria were predominant (87.1%), particularly in females, with *E. coli* accounting for 61.7% of all isolates. This prevalence aligns with global patterns identifying *E. coli*as the primary causative agent of pediatric UTIs, with reported frequencies ranging from 58 to 80% [4, 5, 9]. Also, our study highlighted*E. coli*’s significant role as the predominant causative pathogen of UTIs in both boys and girls, with its prevalence notably higher in girls (69.8%) compared to boys (39.8%). The greater vulnerability of girls to UTIs may be attributed to anatomical and biological differences, notably the shorter urethra in females, which facilitates easier access for enteric organisms to colonize the urinary tract. These findings are consistent with previous studies [1, 2, 4, 5]. Compared to females, males exhibited a higher occurrence of other UTI-causing bacteria like*Klebsiella* spp., *Proteus* spp., *Enterococcus*spp., and other Gram-positive bacteria (P-value < 0.001). This is similar to other study reports [2, 4–6]. Furthermore, the burden of UTIs in this age group may be exacerbated by distinctive environmental and socioeconomic factors in the West Bank, including limited education and awareness regarding hygiene, as well as healthcare resource constraints.

Our findings showed a high prevalence rate of MDR isolates (34.3%) compared to high-income countries but similar rates in low- and middle-income countries [17–20]. Higher rates were previously shown in regional studies 59.9% in Jordan and 66% in Egypt [21, 22]. Although limited data exist on MDR in pediatric UTIs in Palestine, a study from Gaza reported high MDR rates in general, reaching 77.2% in bacterial isolates [23]. The prevalence of ESBL-producing bacteria (33.1%) and CRE (3.3%) further emphasizes the growing challenge of AMR. These rates are consistent with other reports, where ESBL prevalence has ranged from 30 to 40% [24, 25]. Comparable trends have been reported across the region, 46%, 62% in Jordan [26, 27], and 15.5% in Lebanon [28]. At the national level, a study conducted in northern West Bank reported an ESBL prevalence of 38.4% among UTI isolates in patients aged 12 years and above [29]. Similarly, a study from Gaza revealed that 64% were ESBL producers [23]. There are several reasons behind the rise in ESBLs. The overuse of broad-spectrum antibiotics increases the likelihood of bacteria developing resistance, while the widespread availability of over-the-counter antibiotics has led to increased misuse. Moreover, frequent and prolonged hospitalization, along with the use of medical devices such as urinary catheters, has facilitated the transmission of ESBLs. Community spread of ESBLs among otherwise healthy children, particularly those recently exposed to antibiotics, is also becoming increasingly prevalent. All of these factors underscore the importance of using antibiotics responsibly and with caution [30–32]. According to CRE, previous studies from Palestine reported an annual CRE prevalence ranging from 4.3 to 6.28% [33, 34]. Furthermore, children < 2 years exhibited greater increased MDR rates than the older age group, which aligns with different findings indicating that infants and younger pediatrics are at greater risk of infections with drug-resistant strains. This could be due to an immature immune system, higher rates of hospitalization, and frequent antibiotic exposure [35]. Also, the absence of regulations on antibiotic usage and the extensive over-the-counter availability of antibiotics in Palestine substantially exacerbate this issue. The accessibility of antibiotics without a prescription facilitates their misuse and overuse, intensifying the emergence and dissemination of antimicrobial resistance in the area. The high prevalence of MDR and ESBL in the West Bank could be attributed to multiple factors; inappropriately prescribed broad-spectrum antibiotics and unregulated use in hospitals and the community accelerate the emergence of resistance [7, 8, 13, 36, 37].

Regarding MDR rates, among the highest percentage of *Proteus* spp. shows the highest (50.6%), followed by *Klebsiella* spp. (45.4%) and *E. coli* (32.6%). Other studies also showed high rates of MDR among *Proteus*spp. [38]. These results, which show higher MDR rates in*Klebsiella* spp. than *E. coli*, also have been found in another study [39], whereas most of the studies show higher MDR rates in*E. coli*[40]. These discrepancies may result from factors such as geographic variations, differences in antibiotic usage, and clinical settings. Consequently, it is hard to definitively state which organism universally has higher MDR rates. However, it is thought that hospital-acquired*Klebsiella*spp. tends to exhibit MDR [41]. Variation in antimicrobial resistance across hospitals were noticed, many factors contribute to this, including differences in antibiotic prescribing practices, as hospitals with higher antibiotic use showed higher resistance rates. This was especially evident during the COVID-19 pandemic, which coincided with the years during which our study was conducted, when most hospitalized patients received antibiotics, and hospital-acquired resistant infections surged [42–44]. In addition to that, selective antibiotic pressure remains a key driver of resistance emergence through multiple documented cases [45]. Cross-contamination via contaminated equipment or staff hands further contributes to hospital-acquired transmission and local outbreaks [46]. However, hospitals with strong antimicrobial stewardship programs have been shown to achieve notable reductions in resistance rates, demonstrating how institutional policies affect regional resistance patterns [47]. Lastly, differences in patient populations whether in number or type affect both the prevalence and type of resistant bacteria. For example, tertiary care centers, which treat sicker and more immunocompromised patients, correlate with higher resistance prevalence [48, 49]. It is worth mentioning that, according to recent studies, the incidence of pediatric UTIs decreased during the COVID-19 pandemic, which coincides with the period covered by our study. This suggests that if the study had been conducted in different years, outside the pandemic period, the findings could have been even more concerning, as UTI rates were likely suppressed due to COVID-19-related restrictions [50–52].

The American Academy of Pediatrics (AAP) recommends using third-generation cephalosporins empirically as the parenteral choice for hospitalized pediatric patients with UTI and cephalosporins or nitrofurantoin for outpatient therapy [53]. Our study results, with a high decrease in Ampicillin matching (16.8%), and 49.7% and 51.9% for Ceftriaxone and Cefotaxime, respectively, there was substantial resistance to third-generation cephalosporin and empirical antibiotics, demonstrated that ampicillin and TMP/SMT were no longer effective against the most commonly encountered pathogens and a worrying situation regarding the rest of the empirical antibiotics [54, 55].

Study limitations include the inability to involve the Gaza Strip and private hospitals in the West Bank, which may affect the generalizability of the study results to the Palestinian population or introduce the possibility of sampling bias. Also, because the study is cross-sectional and retrospective, several limitations occurred, including the complete reliance on previous electronic records and the absence of molecular studies and gene identification. Furthermore, the unavailability of some important variables related to the patient’s clinical presentation, such as symptoms, disease severity, the presence of other comorbidities, or prior antibiotic use, limited our ability to make clinical correlations and identify risk factors associated with increased resistance. Therefore, establishing causal links between variables was not possible. All these factors may have influenced the resistance characteristics of the antibiotics examined in this investigation. Nevertheless, these findings serve as a valuable reference for decision-makers seeking to implement effective measures and assess antibiotics’ effectiveness in the future. They also highlight regional differences and underline the importance of routinely repeating similar assessments to guide clinical practice.

Since UTIs are among the most common infectious diseases in the pediatric population worldwide, understanding the causative organisms and their antibiotic sensitivity profiles in the West Bank, Palestine, is crucial. Establishing regional antibiograms and enhancing collaboration across Palestine for infection control and antimicrobial stewardship are essential to addressing the growing concerns of antimicrobial resistance (AMR). The development of regional antibiograms is particularly important given the available data and, more significantly, in the context of the ongoing conflict in Palestine, where there is a severe shortage of antibiotics.

## Conclusion

The findings of this study highlight the considerable prevalence of MDR organisms among pediatric patients with UTIs in the West Bank, Palestine. Gram-negative bacteria were the primary pathogens, with *E. coli* and *Klebsiella* spp. being the most commonly isolated species. A high level of resistance was observed, particularly against ampicillin, a commonly prescribed antibiotic. While these results raise concerns regarding the adequacy of current empiric treatment regimens, further studies incorporating clinical outcomes are necessary to better inform treatment decisions and to explore risk factors associated with the emergence and spread of MDR organisms.

## Supplementary Information

Supplementary Material 1.

## Data Availability

The data used to support the findings of this study are included within the article.
