# Supplementary material for: Bacterial etiology and antimicrobial resistance patterns of pediatric UTIs in the West bank, Palestine: a cross-sectional study
Source: BMC Pediatr. 2025 Oct 2;25:725. doi: 10.1186/s12887-025-06054-0 (PMC12492803; doi:10.1186/s12887-025-06054-0)
Supplement: Supplementary file 1 — Supplementary Material 1. [file 12887_2025_6054_MOESM1_ESM.docx]

**Table S1:** Clinically relevant antibiotics resistance of UTI Isolated Bacteria at three tertiary governmental hospitals at West Bank, Paelstine

| **Antibiotics** | **Uropathogenic isolated bacteria and resistance rate to antibiotics** | | | | | | |
| --- | --- | --- | --- | --- | --- | --- | --- |
|  | ***E. coli*** | ***Klebsiella spp.*** | ***P. aeruginosa*** | ***Proteus spp.*** | ***Enterobacter spp.*** | ***Enterococcus spp.*** | ***Staphylococcus aureus*** |
| Amikacin | 20(0.8) | 53(9.1) | 3(2.7) | 1(1.3) | 5(8.1) | - | - |
| Amoxicillin | - | - | - | 9(10.8) | 19(29.7) | - | - |
| Amoxicillin/clavulanic acid | 620(26.3) | 176(31.1) | - | 11(15.3) | 56(88.9) | 26(32.9) | 3(17.6) |
| Ampicillin | 1484(75.9) | 421(98.6) | - | 43(70.5) | 12(100) | 57(30) | - |
| Aztreonam | 6(50.0) | 2(40.0) | - | - | - | - | - |
| Cefazolin | 68(32.2) | 11(61.1) | - | 0(0.00) | 1(100) | - | - |
| Cefdinir | - | - | - | - | - | - | - |
| Cefepime | 695(36.0) | 245(49.6) | 2(2.2) | 7(10.1) | 5(12.5) | - | - |
| Cefixime | 25(15.5) | 6(54.5) | - | - | 1(100) | - | 1(100) |
| Cefotaxime | 905(37.3) | 303(-) | - | - | - | - | - |
| Cefoxitin | - | - | - | - | - | - | 17(39.5) |
| Ceftazidime | 844(37.4) | 289(51.1) | 8(7.3) | 7(8.8) | 16(26.2) | - | - |
| Ceftriaxone | 879(37.7) | 293(50.8) | - | 5(6.8) | - | - | 1(11.1) |
| Cefuroxime | - | - | - | - | - | - | 0(.00) |
| Ciprofloxacin | 479(19.8) | 70(11.7) | 14(12.5) | 13(15.9) | 8(12.5) | 82(37.4) | 9(20.5) |
| Clindamycin | - | - | - | - | - | - | 10(26.3) |
| Colistin | 0(0.00) | 3(5.9) | 0(0.00) | - | 0(0.00) | - | - |
| Ertapenem | 4(0.2) | 61(10.3) | - | 0(0.00) | 3(4.7) | - | - |
| Erythromycin | - | - | - | - | - | - | 12(60.0) |
| Fosfomycin | 7(2.1) | - | - | - | - | - | - |
| Fusidic acid | - | - | - | - | - | - | 2(100) |
| Gentamicin | 363(15.0) | 142(23.8) | 8(7.3) | 4(4.8) | 9(13.8) | 62(58.5) | 2(7.1) |
| Imipenem | 4(0.3) | 11(3.4) | 7(7.1) | 2(5) | 1(2.9) | - | - |
| Levofloxacin | - | 0(0.00) | 11(16.4) | 0(0.00) | 0(0.00) | - | - |
| Meropenem | 5(0.2) | 61(10.3) | 6(5.4) | 0(0.00) | 4(6.1) | - | 0(0.00) |
| Nitrofurantoin | 42(1.8) | 93(16.5) | 75(96.2) | - | 7(11.1) | 9(4.1) | 1(2.6) |
| Penicillin | - | - | 5(16.7) | - | - | - | - |
| Piperacillin / Tazobactam | 107(4.4) | 96(16.4) | 4(3.7) | 0(0.00) | - | - | - |
| Rifampicin | - | - | - | - | - | - | 1(8.3) |
| Teicoplanim | - | - | - | - | - | 1(2.1) | 0(0.00) |
| Tigecycline | - | - | - | - | - | - | - |
| Trimethoprim/ Sulfamethoxazole | 1144(47.3) | 277(46.9) | - | 44(53.7) | 11(17.7) | - | 5(12.8) |
| Vancomycin | - | - | - | 0(0.00) | - | 3(1.3) | 0(0.00) |
|  |  |  |  |  |  |  |  |
|  |  |  |  |  |  |  |  |
|  |  |  |  |  |  |  |  |
|  |  |  |  |  |  |  |  |
